# Supplementary material for: Spatial sequestration and detoxification of Huntingtin by the ribosome quality control complex
Source: eLife. 2016 Apr 1;5:e11792. doi: 10.7554/eLife.11792 (PMC4868537; doi:10.7554/eLife.11792)
Supplement: Supplementary file 1. — DOI: http://dx.doi.org/10.7554/eLife.11792.015 [file elife-11792-supp1.pptx]

## Slide 1
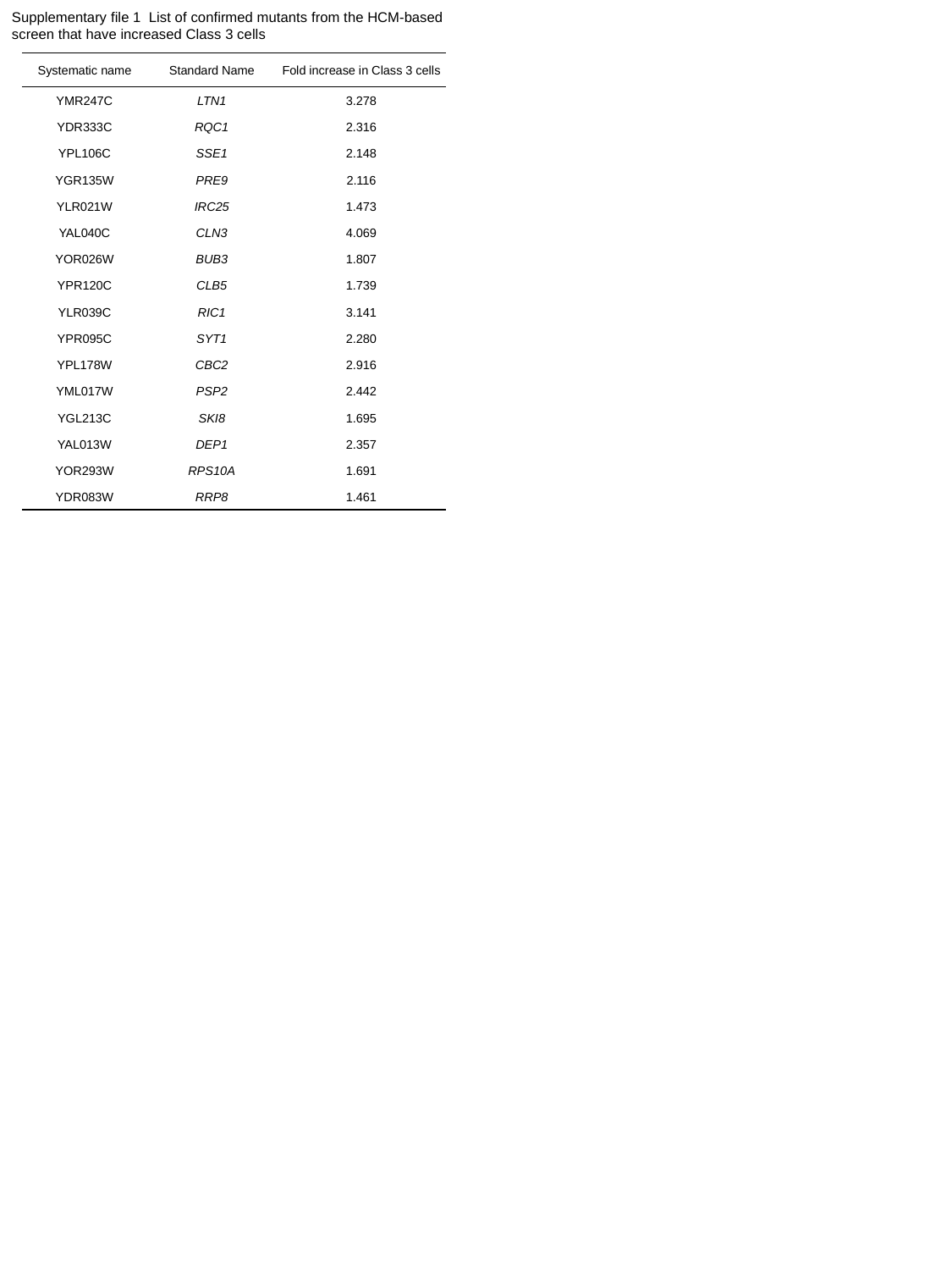

Supplementary file 1 List of confirmed mutants from the HCM-based
screen that have increased Class 3 cells
| Systematic name | Standard Name | Fold increase in Class 3 cells |
| --- | --- | --- |
| YMR247C | LTN1 | 3.278 |
| YDR333C | RQC1 | 2.316 |
| YPL106C | SSE1 | 2.148 |
| YGR135W | PRE9 | 2.116 |
| YLR021W | IRC25 | 1.473 |
| YAL040C | CLN3 | 4.069 |
| YOR026W | BUB3 | 1.807 |
| YPR120C | CLB5 | 1.739 |
| YLR039C | RIC1 | 3.141 |
| YPR095C | SYT1 | 2.280 |
| YPL178W | CBC2 | 2.916 |
| YML017W | PSP2 | 2.442 |
| YGL213C | SKI8 | 1.695 |
| YAL013W | DEP1 | 2.357 |
| YOR293W | RPS10A | 1.691 |
| YDR083W | RRP8 | 1.461 |
