# Supplementary material for: Spatial sequestration and detoxification of Huntingtin by the ribosome quality control complex
Source: eLife. 2016 Apr 1;5:e11792. doi: 10.7554/eLife.11792 (PMC4868537; doi:10.7554/eLife.11792)
Supplement: Supplementary file 2. — DOI: http://dx.doi.org/10.7554/eLife.11792.016 [file elife-11792-supp2.pptx]

## Slide 1
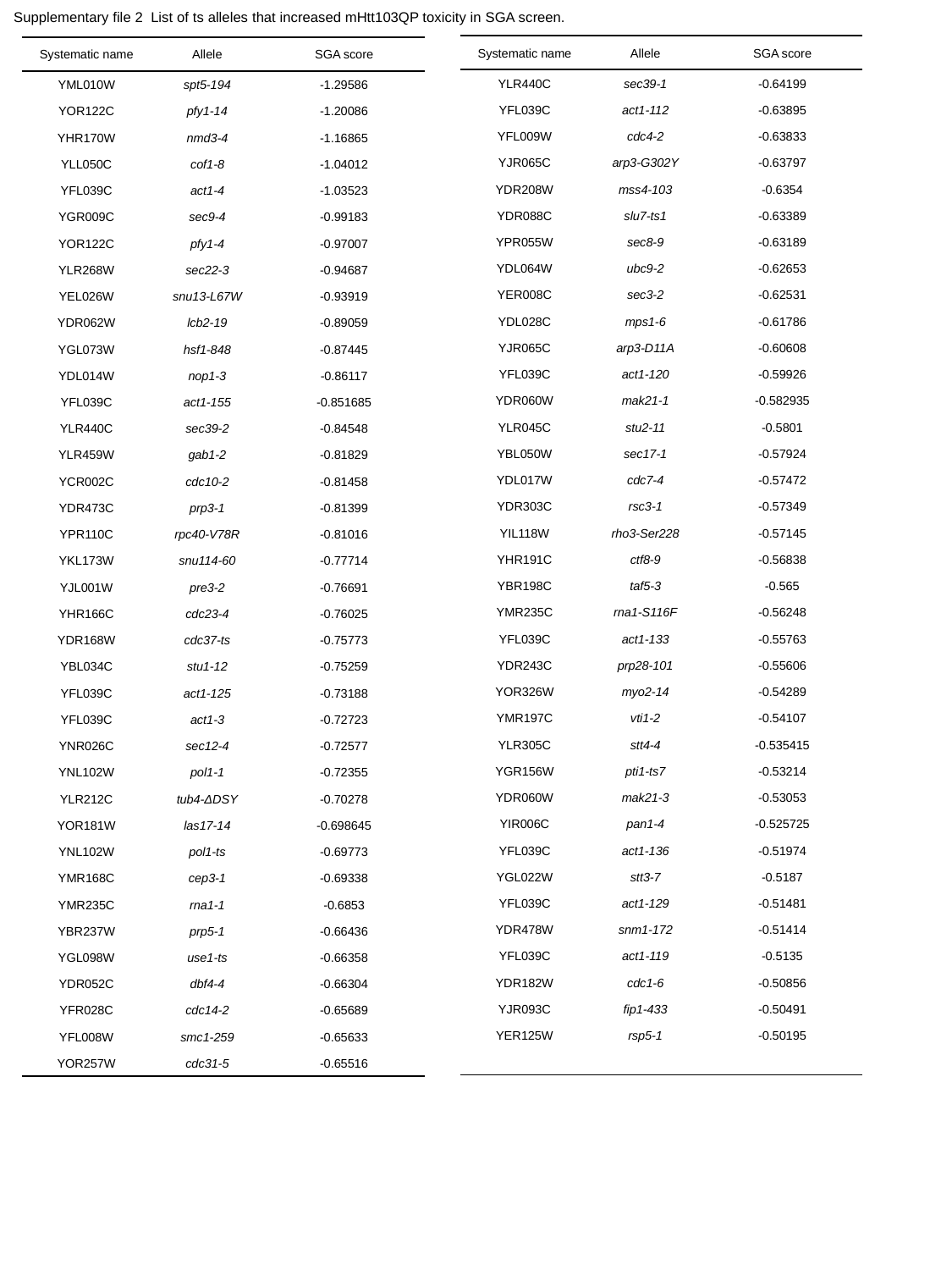

Supplementary file 2 List of ts alleles that increased mHtt103QP toxicity in SGA screen.
| Systematic name | Allele | SGA score |
| --- | --- | --- |
| YLR440C | sec39-1 | -0.64199 |
| YFL039C | act1-112 | -0.63895 |
| YFL009W | cdc4-2 | -0.63833 |
| YJR065C | arp3-G302Y | -0.63797 |
| YDR208W | mss4-103 | -0.6354 |
| YDR088C | slu7-ts1 | -0.63389 |
| YPR055W | sec8-9 | -0.63189 |
| YDL064W | ubc9-2 | -0.62653 |
| YER008C | sec3-2 | -0.62531 |
| YDL028C | mps1-6 | -0.61786 |
| YJR065C | arp3-D11A | -0.60608 |
| YFL039C | act1-120 | -0.59926 |
| YDR060W | mak21-1 | -0.582935 |
| YLR045C | stu2-11 | -0.5801 |
| YBL050W | sec17-1 | -0.57924 |
| YDL017W | cdc7-4 | -0.57472 |
| YDR303C | rsc3-1 | -0.57349 |
| YIL118W | rho3-Ser228 | -0.57145 |
| YHR191C | ctf8-9 | -0.56838 |
| YBR198C | taf5-3 | -0.565 |
| YMR235C | rna1-S116F | -0.56248 |
| YFL039C | act1-133 | -0.55763 |
| YDR243C | prp28-101 | -0.55606 |
| YOR326W | myo2-14 | -0.54289 |
| YMR197C | vti1-2 | -0.54107 |
| YLR305C | stt4-4 | -0.535415 |
| YGR156W | pti1-ts7 | -0.53214 |
| YDR060W | mak21-3 | -0.53053 |
| YIR006C | pan1-4 | -0.525725 |
| YFL039C | act1-136 | -0.51974 |
| YGL022W | stt3-7 | -0.5187 |
| YFL039C | act1-129 | -0.51481 |
| YDR478W | snm1-172 | -0.51414 |
| YFL039C | act1-119 | -0.5135 |
| YDR182W | cdc1-6 | -0.50856 |
| YJR093C | fip1-433 | -0.50491 |
| YER125W | rsp5-1 | -0.50195 |
| | | |
| Systematic name | Allele | SGA score |
| --- | --- | --- |
| YML010W | spt5-194 | -1.29586 |
| YOR122C | pfy1-14 | -1.20086 |
| YHR170W | nmd3-4 | -1.16865 |
| YLL050C | cof1-8 | -1.04012 |
| YFL039C | act1-4 | -1.03523 |
| YGR009C | sec9-4 | -0.99183 |
| YOR122C | pfy1-4 | -0.97007 |
| YLR268W | sec22-3 | -0.94687 |
| YEL026W | snu13-L67W | -0.93919 |
| YDR062W | lcb2-19 | -0.89059 |
| YGL073W | hsf1-848 | -0.87445 |
| YDL014W | nop1-3 | -0.86117 |
| YFL039C | act1-155 | -0.851685 |
| YLR440C | sec39-2 | -0.84548 |
| YLR459W | gab1-2 | -0.81829 |
| YCR002C | cdc10-2 | -0.81458 |
| YDR473C | prp3-1 | -0.81399 |
| YPR110C | rpc40-V78R | -0.81016 |
| YKL173W | snu114-60 | -0.77714 |
| YJL001W | pre3-2 | -0.76691 |
| YHR166C | cdc23-4 | -0.76025 |
| YDR168W | cdc37-ts | -0.75773 |
| YBL034C | stu1-12 | -0.75259 |
| YFL039C | act1-125 | -0.73188 |
| YFL039C | act1-3 | -0.72723 |
| YNR026C | sec12-4 | -0.72577 |
| YNL102W | pol1-1 | -0.72355 |
| YLR212C | tub4-ΔDSY | -0.70278 |
| YOR181W | las17-14 | -0.698645 |
| YNL102W | pol1-ts | -0.69773 |
| YMR168C | cep3-1 | -0.69338 |
| YMR235C | rna1-1 | -0.6853 |
| YBR237W | prp5-1 | -0.66436 |
| YGL098W | use1-ts | -0.66358 |
| YDR052C | dbf4-4 | -0.66304 |
| YFR028C | cdc14-2 | -0.65689 |
| YFL008W | smc1-259 | -0.65633 |
| YOR257W | cdc31-5 | -0.65516 |
