# Supplementary material for: Spatial sequestration and detoxification of Huntingtin by the ribosome quality control complex
Source: eLife. 2016 Apr 1;5:e11792. doi: 10.7554/eLife.11792 (PMC4868537; doi:10.7554/eLife.11792)
Supplement: Supplementary file 3. — A. List of S. cerevisiae strains. B. List of plasmids DOI: http://dx.doi.org/10.7554/eLife.11792.017 [file elife-11792-supp3.pptx]

## Slide 1
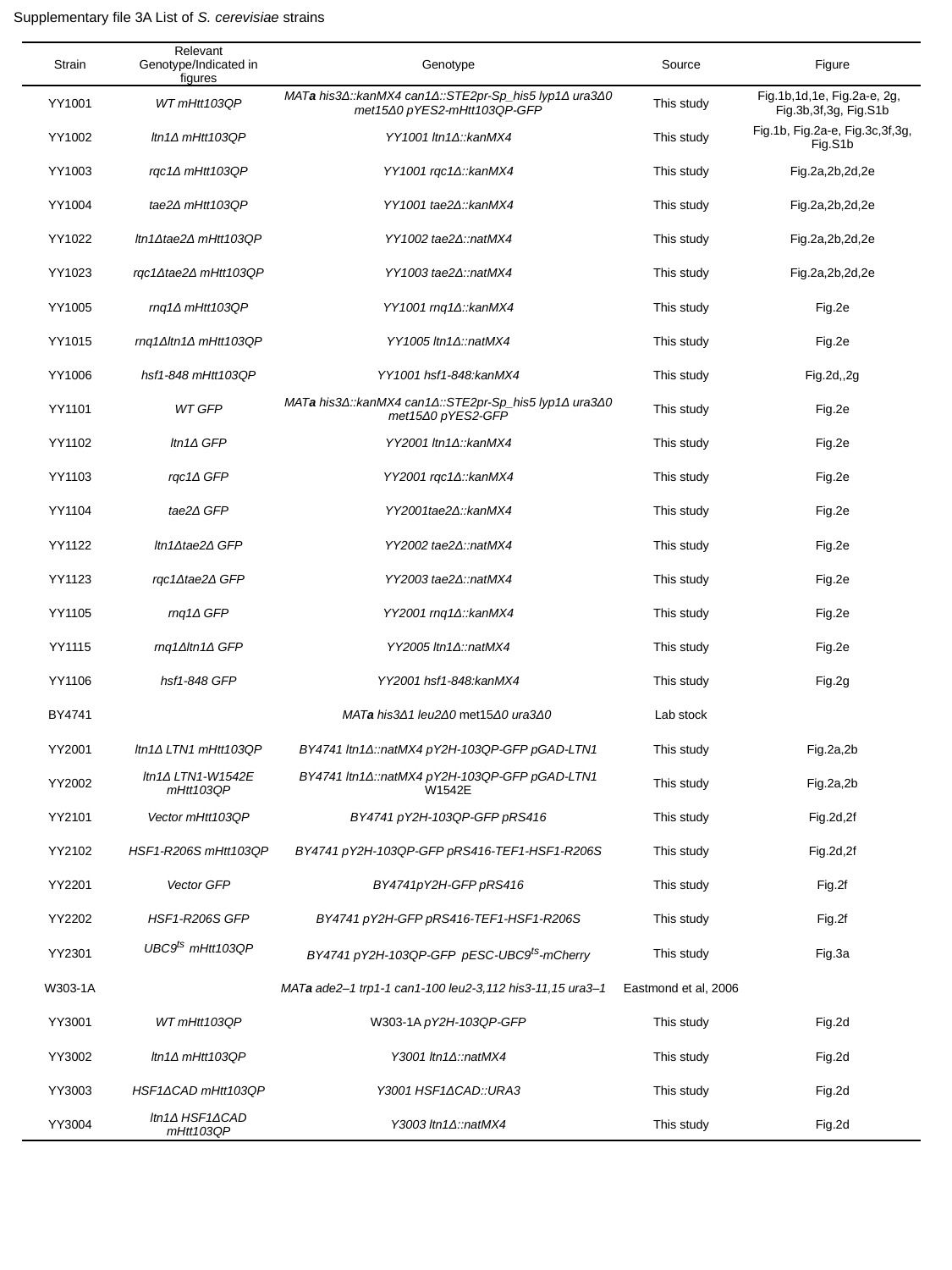

Supplementary file 3A List of S. cerevisiae strains
| Strain | Relevant Genotype/Indicated in figures | Genotype | Source | Figure |
| --- | --- | --- | --- | --- |
| YY1001 | WT mHtt103QP | MATa his3Δ::kanMX4 can1Δ::STE2pr-Sp\_his5 lyp1Δ ura3Δ0 met15Δ0 pYES2-mHtt103QP-GFP | This study | Fig.1b,1d,1e, Fig.2a-e, 2g, Fig.3b,3f,3g, Fig.S1b |
| YY1002 | ltn1Δ mHtt103QP | YY1001 ltn1Δ::kanMX4 | This study | Fig.1b, Fig.2a-e, Fig.3c,3f,3g, Fig.S1b |
| YY1003 | rqc1Δ mHtt103QP | YY1001 rqc1Δ::kanMX4 | This study | Fig.2a,2b,2d,2e |
| YY1004 | tae2Δ mHtt103QP | YY1001 tae2Δ::kanMX4 | This study | Fig.2a,2b,2d,2e |
| YY1022 | ltn1Δtae2Δ mHtt103QP | YY1002 tae2Δ::natMX4 | This study | Fig.2a,2b,2d,2e |
| YY1023 | rqc1Δtae2Δ mHtt103QP | YY1003 tae2Δ::natMX4 | This study | Fig.2a,2b,2d,2e |
| YY1005 | rnq1Δ mHtt103QP | YY1001 rnq1Δ::kanMX4 | This study | Fig.2e |
| YY1015 | rnq1Δltn1Δ mHtt103QP | YY1005 ltn1Δ::natMX4 | This study | Fig.2e |
| YY1006 | hsf1-848 mHtt103QP | YY1001 hsf1-848:kanMX4 | This study | Fig.2d,,2g |
| YY1101 | WT GFP | MATa his3Δ::kanMX4 can1Δ::STE2pr-Sp\_his5 lyp1Δ ura3Δ0 met15Δ0 pYES2-GFP | This study | Fig.2e |
| YY1102 | ltn1Δ GFP | YY2001 ltn1Δ::kanMX4 | This study | Fig.2e |
| YY1103 | rqc1Δ GFP | YY2001 rqc1Δ::kanMX4 | This study | Fig.2e |
| YY1104 | tae2Δ GFP | YY2001tae2Δ::kanMX4 | This study | Fig.2e |
| YY1122 | ltn1Δtae2Δ GFP | YY2002 tae2Δ::natMX4 | This study | Fig.2e |
| YY1123 | rqc1Δtae2Δ GFP | YY2003 tae2Δ::natMX4 | This study | Fig.2e |
| YY1105 | rnq1Δ GFP | YY2001 rnq1Δ::kanMX4 | This study | Fig.2e |
| YY1115 | rnq1Δltn1Δ GFP | YY2005 ltn1Δ::natMX4 | This study | Fig.2e |
| YY1106 | hsf1-848 GFP | YY2001 hsf1-848:kanMX4 | This study | Fig.2g |
| BY4741 | | MATa his3Δ1 leu2Δ0 met15Δ0 ura3Δ0 | Lab stock | |
| YY2001 | ltn1Δ LTN1 mHtt103QP | BY4741 ltn1Δ::natMX4 pY2H-103QP-GFP pGAD-LTN1 | This study | Fig.2a,2b |
| YY2002 | ltn1Δ LTN1-W1542E mHtt103QP | BY4741 ltn1Δ::natMX4 pY2H-103QP-GFP pGAD-LTN1 W1542E | This study | Fig.2a,2b |
| YY2101 | Vector mHtt103QP | BY4741 pY2H-103QP-GFP pRS416 | This study | Fig.2d,2f |
| YY2102 | HSF1-R206S mHtt103QP | BY4741 pY2H-103QP-GFP pRS416-TEF1-HSF1-R206S | This study | Fig.2d,2f |
| YY2201 | Vector GFP | BY4741pY2H-GFP pRS416 | This study | Fig.2f |
| YY2202 | HSF1-R206S GFP | BY4741 pY2H-GFP pRS416-TEF1-HSF1-R206S | This study | Fig.2f |
| YY2301 | UBC9ts mHtt103QP | BY4741 pY2H-103QP-GFP pESC-UBC9ts-mCherry | This study | Fig.3a |
| W303-1A | | MATa ade2–1 trp1-1 can1-100 leu2-3,112 his3-11,15 ura3–1 | Eastmond et al, 2006 | |
| YY3001 | WT mHtt103QP | W303-1A pY2H-103QP-GFP | This study | Fig.2d |
| YY3002 | ltn1Δ mHtt103QP | Y3001 ltn1Δ::natMX4 | This study | Fig.2d |
| YY3003 | HSF1ΔCAD mHtt103QP | Y3001 HSF1ΔCAD::URA3 | This study | Fig.2d |
| YY3004 | ltn1Δ HSF1ΔCAD mHtt103QP | Y3003 ltn1Δ::natMX4 | This study | Fig.2d |

## Slide 2
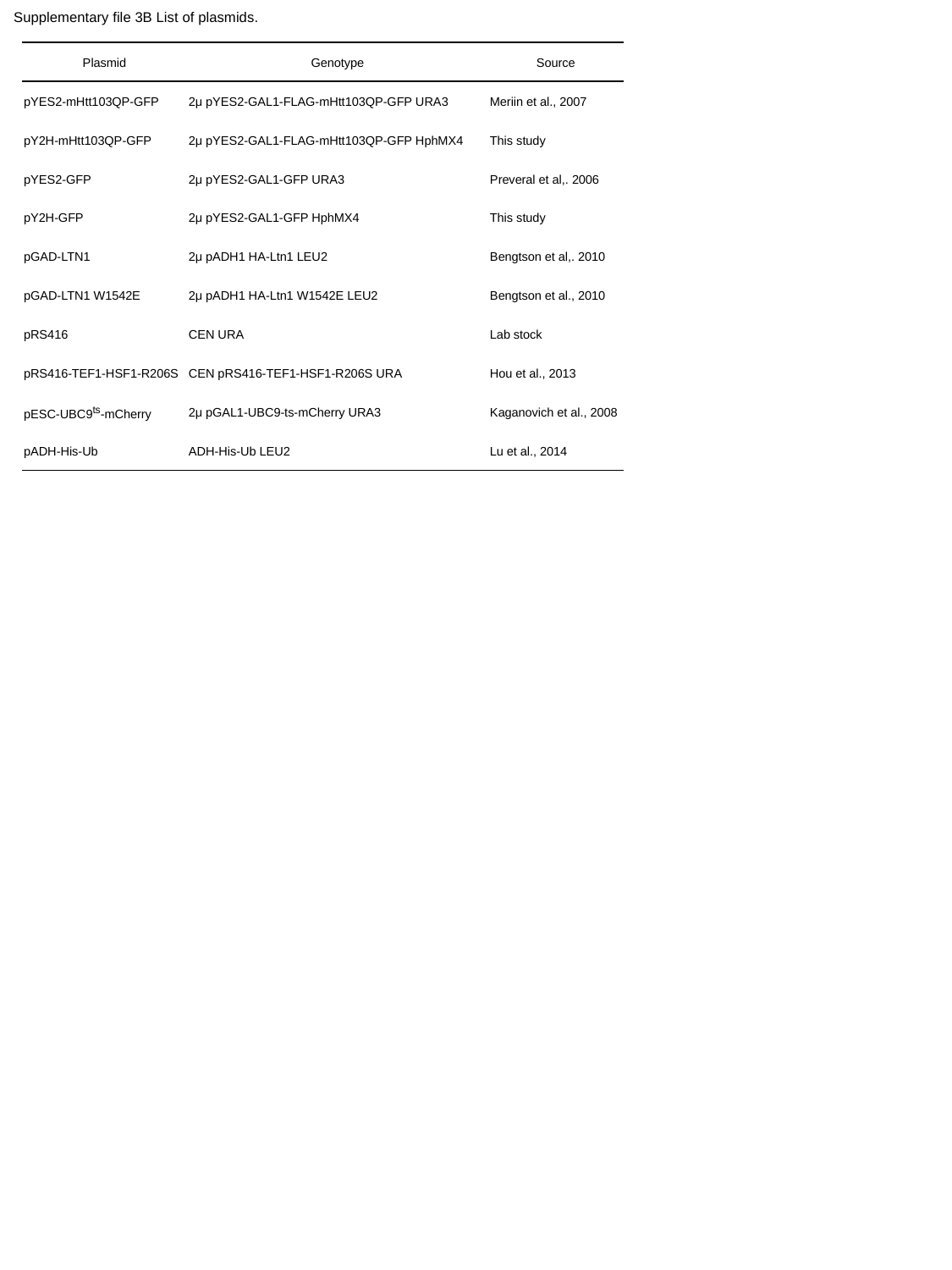

Supplementary file 3B List of plasmids.
| Plasmid | Genotype | Source |
| --- | --- | --- |
| pYES2-mHtt103QP-GFP | 2μ pYES2-GAL1-FLAG-mHtt103QP-GFP URA3 | Meriin et al., 2007 |
| pY2H-mHtt103QP-GFP | 2μ pYES2-GAL1-FLAG-mHtt103QP-GFP HphMX4 | This study |
| pYES2-GFP | 2μ pYES2-GAL1-GFP URA3 | Preveral et al,. 2006 |
| pY2H-GFP | 2μ pYES2-GAL1-GFP HphMX4 | This study |
| pGAD-LTN1 | 2μ pADH1 HA-Ltn1 LEU2 | Bengtson et al,. 2010 |
| pGAD-LTN1 W1542E | 2μ pADH1 HA-Ltn1 W1542E LEU2 | Bengtson et al., 2010 |
| pRS416 | CEN URA | Lab stock |
| pRS416-TEF1-HSF1-R206S | CEN pRS416-TEF1-HSF1-R206S URA | Hou et al., 2013 |
| pESC-UBC9ts-mCherry | 2μ pGAL1-UBC9-ts-mCherry URA3 | Kaganovich et al., 2008 |
| pADH-His-Ub | ADH-His-Ub LEU2 | Lu et al., 2014 |
